# 895. Impact of Telemedicine on HIV Care and Prevention Services at an Academic Ryan White-Funded Clinic

**DOI:** 10.1093/ofid/ofab466.1090

**Published:** 2021-12-04

**Authors:** Jay V Dasigi, Nupur Gupta, Christiane Hadi

**Affiliations:** 1 Universirty of Pittsburgh Medical Center, Pittsburgh, Pennsylvania; 2 University of Pittsburgh Medical Center, Pittsburgh, Pennsylvania

## Abstract

**Background:**

Telemedicine (TM) has been seldom used for the care of persons with HIV. However, the COVID-19 pandemic has forced HIV clinics to rapidly scale TM resources. With the increase of TM, the impact on HIV patient care remains uncertain. The purpose of this study is to examine the effects of TM on HIV care and retention at a Ryan White-funded clinic.

**Methods:**

This was a retrospective study of patients seen at an academic clinic in Pittsburgh, PA between 1/1/20 – 12/31/20. Encounter information was extracted from the clinic electronic health record. Primary outcomes were viral load (VL) suppression (< 200 copies/ml) and retention in care for persons seen via TM (phone, video +/- in person) vs those seen in-person. Secondary outcomes included flu vaccination and STI screening rates.

**Results:**

Amongst 1414 patients, 608 patients had at least one scheduled TM visit, with 97 seen exclusively via TM, and 806 were scheduled for only in-person visits. In those with at least one TM visit, 92.72% had a suppressed VL. 89.69% of those with only TM visits were suppressed. 92.43% were suppressed in the in-person group. Average show rate amongst patients who had at least one TM visit was 60.39% (+0.96% from 2019, +1.71% from 2018), vs 64.38% amongst patients who only had in-person visits. Amongst patients who were only scheduled for TM visits, show rate was 83.97%. 40.18% of patients who had at least one TM visit received their flu vaccine in 2020 (-37.45% from 2019, -36.72% from 2018) vs 37.62% who were only seen in-person. Amongst patients who had at least one TM visit, syphilis screening rate was 43.09% (-7.64% from 2019, -8.55% from 2018) vs 43.51% for those seen only in-person. Gonorrhea and chlamydia screening rates were both 42.91% (+9.46% from 2019, +15.27% from 2018 for chlamydia screening; +8.36% from 2019, +14.73% from 2018 for gonorrhea screening). Amongst patients who were exclusively seen in-person gonorrhea screening rate was 48.24% and chlamydia screening rate was 47.57%.

Table 1. Characteristics of Patients Seen in 2020

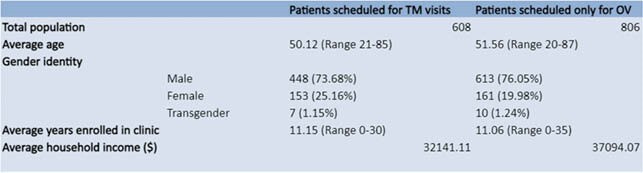

Table 2. Primary and Secondary Outcomes for Patients Seen in 2020

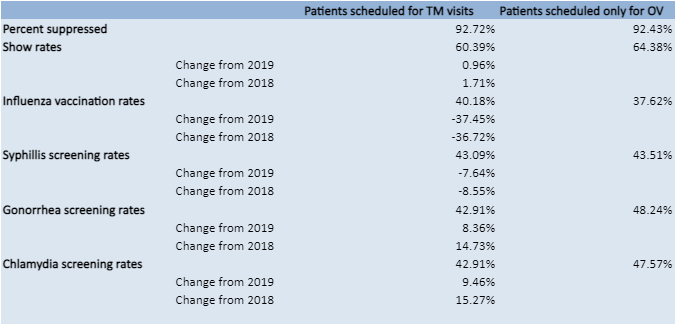

**Conclusion:**

VL suppression rates were similar across both groups, but retention in care was highest in the TM-only group. Flu vaccination rates and STI screening were lower in the groups that included TM. TM is an effective method for maintaining VL suppression and retention in care but has room for improvement with provision of preventative services.

**Disclosures:**

**All Authors**: No reported disclosures

